# Quality of life and self-regulatory fatigue in patients with type 2 diabetes mellitus: the mediating effects of personal mastery and health-promoting behaviors

**DOI:** 10.3389/fmed.2026.1835765

**Published:** 2026-05-12

**Authors:** Guiqiu Fang, Weidong Gong, Lihua Xia, Liping Li, Peng Sun, Jie Song, Yufeng Cui, Xiaochun Tang, Youxing Wu, Panfeng Li, Xiaoyan Zhang, Xing Ni, Feng Lu, Jiangyun Ru, Jun Yin, Yuanzhi Li, Zhangyi Wang

**Affiliations:** 1 Department of Nursing, Tianjin Fourth Central Hospital, Tianjin, China; 2 Gastrointestinal Surgery/National Key Clinical Construction Specialist General Surgery, Affiliated Hengyang Hospital of Hunan Normal University and Hengyang Central Hospital, Hengyang, Hunan, China; 3 Department of Deanery, Tianjin First Central Hospital, Tianjin, China

**Keywords:** health-promoting behaviors, personal mastery, quality of life, self-regulatory fatigue, structural equation modeling, type 2 diabetes mellitus

## Abstract

**Background:**

Quality of life (QoL) is a key prognostic indicator in type 2 diabetes mellitus (T2DM). The interplay between psychological and behavioral determinants remains underexplored.

**Objectives:**

To examine QoL and its associated factors in T2DM patients, and to test the mediating roles of personal mastery (PM) and health-promoting behaviors (HPB) between self-regulatory fatigue (SRF) and QoL.

**Methods:**

A cross-sectional study recruited 432 T2DM patients from three Chinese tertiary hospitals (July 2025–February 2026). Data were collected using the Demographic Characteristics Questionnaire, Self-Regulatory Fatigue Scale, Personal Mastery Scale, Diabetes Health Promotion Scale, and Diabetes-Specific Quality of Life Scale. Pearson’s correlation, multiple linear regression, and structural equation modeling were employed for data analysis. The mediating effects were tested using the bootstrap method.

**Results:**

The total scores for SRF, PM, HPB, and QoL were 38.63 ± 12.84, 26.12 ± 7.62, 97.69 ± 20.87, and 69.41 ± 20.89, respectively. QoL correlated positively with SRF (*r* = 0.581) and negatively with PM (*r* = −0.557) and HPB (*r* = −0.613, all *p* < 0.01). Ten independent predictors of QoL were identified (age, educational level, medical insurance payment method, smoking status, glycemic control status, number of comorbidities, self-care ability, SRF, PM, HPB; *R*^2^ = 0.418). SRF showed a total indirect effect on QoL via PM (28.7%) and HPB (44.6%), plus a chain mediation via PM→HPB (11.3%).

**Conclusion:**

SRF is associated with lower QoL in T2DM patients, partly through reduced PM and HPB. Interventions targeting self-regulatory resources and PM may improve QoL. In parallel, strengthening personal mastery through empowerment-based education, goal setting, and individualized feedback can enhance patients’ confidence in diabetes self-management, which may promote sustained engagement in healthy behaviors and ultimately improve QoL in this population.

## 1 Introduction

Type 2 diabetes mellitus (T2DM) is a chronic metabolic disorder requiring lifelong self-management. The disease places substantial demands on patients’ regulatory resources, potentially impacting their quality of life (QoL) (1, 2). While the physiological complications of T2DM are well-documented, the interplay between psychological and behavioral determinants of QoL remains underexplored.

China currently has the largest diabetic population worldwide, with T2DM representing the predominant form. According to recent epidemiological data, approximately 148 million adults in China were living with diabetes in 2024, corresponding to a prevalence of 11.2%. The disease exhibits a gender disparity, with higher prevalence observed in men than in women (3). The economic implications are substantial; global direct health expenditure on diabetes was estimated at USD 825 billion annually, and China’s share is projected to reach USD 170 billion by 2025, positioning China as the country most economically burdened by diabetes globally (4). Moreover, as highlighted in the Guidelines for the Prevention and Treatment of Diabetes in China (2024 Edition) (5), the disease burden is escalating exponentially, imposing mounting economic pressure on families, healthcare systems, and society at large.

Quality of life refers to an individual’s subjective perception of their life situation, framed within the context of their cultural and value systems (6). It is a multidimensional construct encompassing physical health, psychological state, level of independence, social relationships, and personal beliefs. Evidence suggests that enhancing QoL can facilitate patients’ reintegration into normal life and society, and promote a more proactive attitude toward disease self-management (7). Higher QoL has been associated with improved emotional wellbeing, greater adherence to treatment regimens, reduced risk of diabetes-related complications, and delayed disease progression (8).

The construct of SRF describes the depletion of self-regulatory resources following repeated or sustained efforts to manage stress or difficult situations, based on the premise that such resources are limited (9). In the context of T2DM, where patients must continually regulate their behavior to maintain glycemic control, SRF may impair their capacity to sustain health-promoting routines, thereby contributing to poor clinical outcomes. SRF can exacerbate diabetes-related impairments across physiological, cognitive, behavioral, and social domains, potentially compounding fatigue and diminishing QoL.

Psychological resource reflects the extent to which individuals perceive themselves as having control over events and outcomes that matter in their lives (10). As a core psychological resource, PM enables individuals to cope more effectively with stress and chronic illness. In the context of T2DM, higher levels of personal mastery have been linked to greater engagement in standardized self-care behaviors, improved glycemic stability, reduced risk of complications, enhanced physical comfort, and, consequently, better QoL (11).

Health-promoting behaviors are proactive measures individuals undertake to maintain or improve their health (12). These include adherence to prescribed medications, dietary modification, regular physical activity, stress management, and routine health monitoring. In T2DM patients, consistent engagement in HPB contributes directly to metabolic control, lowers the likelihood of complications, and enhances both physiological and psychological wellbeing.

While previous studies have independently established the relationships between SRF and QoL, PM and self-management, or HPB and metabolic control, the sequential, causal-chain mechanism linking all four constructs remains largely untested. Specifically, no research has investigated whether PM acts as a “meta-resource” that, once depleted by SRF, subsequently compromises HPB, leading to reduced QoL. Drawing upon the Conservation of Resources (COR) theory and the Theory of Planned Behavior, we propose a hierarchical depletion model. This model posits that SRF first erodes a higher-order psychological resource (PM), which in turn leads to a decline in specific behavioral actions (HPB), ultimately worsening QoL. To address this gap, the present study employed a cross-sectional design to: (1) investigate the status of QoL and its associated factors among patients with T2DM, and (2) examine the mediating effects of PM and HPB in the relationship between SRF and QoL by using structural equation modeling.

**FIGURE 1:**
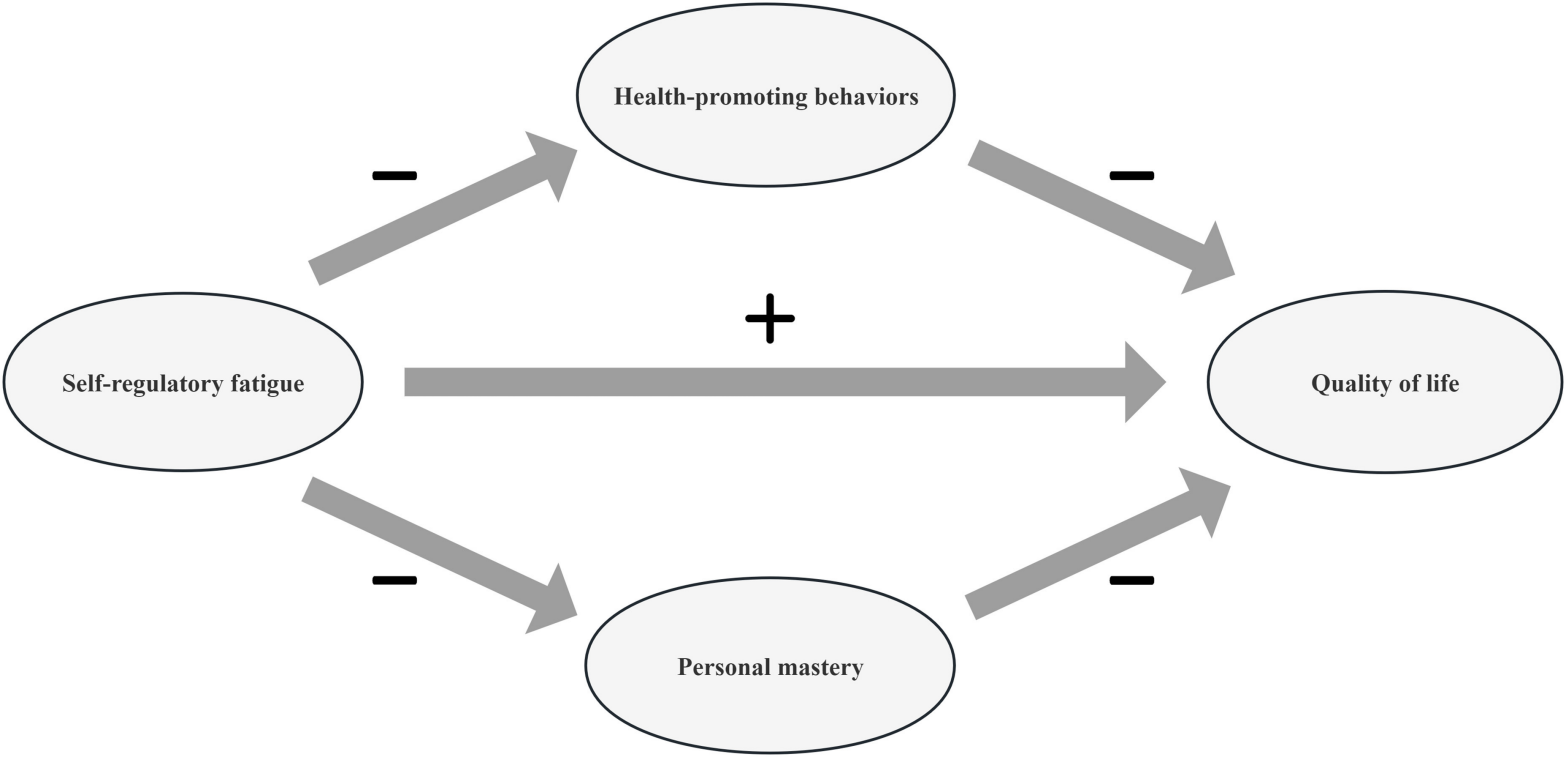
The conceptual framework of self-regulatory fatigue, personal mastery, health-promoting behaviors, and quality of life of the study.

To address this gap, the present study had two primary objectives: (1) to investigate the current status and associated factors of QoL among patients with T2DM, and (2) to examine the mediating roles of PM and HPB in the relationship between SRF and QoL using structural equation modeling. The conceptual framework, informed by the Conservation of Resources theory and Roy’s adaptation model, is presented in Figure 1.

## 2 Materials and methods

### 2.1 Study design and setting

This study used a descriptive cross-sectional design and was conducted in China. The quality reporting of the study adhered to the Strengthening the Reporting of Observational Studies in Epidemiology (STROBE) guidelines, which outline the necessary information to include in reports of cross-sectional studies.

### 2.2 Participants and sample

#### 2.2.1 Sampling procedure and recruitment

A convenience sampling strategy was employed. From July 2025 to February 2026, trained research nurses approached potential participants during their routine outpatient visits or hospitalizations at the endocrinology departments of three tertiary grade-A hospitals in Tianjin and Hengyang, China. The head nurses of each department generated a preliminary list of patients who met the inclusion criteria. Research nurses then sequentially invited these patients in a private setting, explained the study’s aims, assured confidentiality, and obtained written informed consent. Data collection took place in a quiet room adjacent to the clinic to minimize distractions and ensure privacy.

The inclusion criteria were as follows: (1) confirmed diagnosis of T2DM based on American Diabetes Association (ADA) criteria (13); (2) age ≥ 18 years; (3) disease duration ≥ 6 months; (4) ability to read and complete the questionnaires independently or with researcher assistance; and (5) provision of written informed consent. Exclusion criteria: (1) presence of severe diabetes-related complications (e.g., dialysis-dependent nephropathy, proliferative retinopathy, or history of lower extremity amputation) or life-threatening comorbidities (e.g., end-stage cancer, severe heart failure) that could interfere with survey completion or QoL; (2) documented psychiatric disorders or significant cognitive impairment affecting capacity to provide consent or complete the questionnaire; or (3) current participation in other interventional clinical trials.

G*Power 3.1.9.7 software (14) was used to calculate the minimum sample size required for F-tests. A priori power analysis was conducted for linear multiple regression: Fixed model, R^2^ deviation from zero. The parameters were set as follows: effect size f^2^ = 0.15 (medium effect), α err prob = 0.05, and power (1−β err prob) = 0.95. The calculated minimum required sample size was 107.

Considering the complexity of the multi-center design and the structural equation modeling (SEM), we recruited 432 participants from three tertiary grade-A hospitals to ensure adequate statistical power and generalizability. A *post hoc* power analysis using the achieved sample size (*n* = 432), effect size *f*^2^ = 0.15, and α = 0.05 yielded a power (1−β) of 1.00, indicating that our sample size was more than sufficient to detect the hypothesized effects. In addition, participants were recruited from 3 tertiary grade-A hospitals in China through questionnaires, and collected additional questionnaires to enhance the generalizability of the research findings, so as to minimize the deviation of the results caused by geography and hospital nature. In this way, a total of 451 questionnaires were distributed. After eliminating 19 questionnaires with common or obviously contradictory responses, we obtained 432valid questionnaires. This resulted in an effective recovery rate of 95.8%.

### 2.3 Measurements

#### 2.3.1 Demographic characteristics questionnaire

The demographic characteristics questionnaire was developed by the research team to collect a comprehensive set of participant information. It was reviewed for face validity by three senior clinical nurses and two diabetes specialists, but no formal psychometric validation was conducted. It included variables such as gender, age, body mass index (BMI), educational level, marital status, living conditions, residence, employment status, per capita monthly income, medical insurance payment method, drinking status, smoking status, disease duration, number of comorbidities, treatment modality, glycemic control status, whether combined with other chronic diseases, and self-care ability.

#### 2.3.2 Self-regulatory fatigue scale (SRFS)

The Chinese version (15) of the scale originally developed by Nes et al. (16) was employed in this study. This 16-item instrument assesses SRF across three dimensions—cognitive, emotional, and behavioral control—using a five-point Likert scale ranging from 1 (strongly disagree) to 5 (strongly agree). The total possible scores range from 16 to 80, with higher scores reflecting greater levels of SRF. In the present study, the scale demonstrated satisfactory internal consistency, with a Cronbach’s α coefficient of 0.857.

#### 2.3.3 Personal Mastery Scale (PMS)

The Personal Mastery Scale (PMS) was used to assess participants’ perceived control over their lives. The original scale was developed by Pearlin (17). The Chinese version was adapted and validated by Yu et al. (18). We utilized the Chinese version of the scale, which was adapted and validated for the Chinese context by Yu et al. (18). This unidimensional scale comprises seven items, each rated on a 5-point Likert scale ranging from 1 (not at all consistent) to 5 (extremely consistent). Total scores range from 7 to 35, with higher scores reflecting a greater sense of PM. In the present study, the scale demonstrated satisfactory internal consistency, with a Cronbach’s α coefficient of 0.886.

#### 2.3.4 Diabetes health promotion scale (DHPS)

The Chinese version of the scale (19), adapted from the original work by Chen et al. (20), was utilized in this study. This 28-item instrument assesses health-related behaviors across six distinct dimensions: exercise, stress management, health responsibility, risk avoidance, healthy diet, and life appreciation. Each item is rated on a five-point Likert scale ranging from 1 (never) to 5 (always), based on the frequency of engaging in each behavior. The total possible scores range from 28 to 140, with higher scores reflecting more positive and frequent engagement in HPB. The scale demonstrated excellent internal consistency in the current sample, with a Cronbach’s α coefficient of 0.915.

#### 2.3.5 Diabetes-specific quality of life scale (dS-qoLS)

Developed by Fang et al. (21), this 27-item instrument evaluates QoL across four multidimensional domains: physical function, psychological/spiritual status, social relations, and treatment. Each item is rated on a five-point Likert scale reflecting severity, ranging from 1 (none or no problem) to 5 (most severe or extreme difficulty). Total possible scores range from 27 to 135, with higher scores reflecting poorer QoL. In the current study, the scale demonstrated excellent internal consistency, with a Cronbach’s α coefficient of 0.922.

### 2.4 Data collection

Data collection procedures followed the sampling strategy described in section “2.2.1 Sampling procedure and recruitment.” Research nurses provided standardized instructions and remained neutral without guiding participants’ answers. Participants completed the questionnaires independently in a quiet, private room to minimize external distractions. In this study, a total of 451 questionnaires were collected within the specified time, and after excluding 19 invalid questionnaires, a total of 432 valid questionnaires were obtained, with an effective recovery rate of 95.8%.

### 2.5 Statistical analysis

Two researchers independently recorded and analyzed the raw data using Epidata 3.1, IBM SPSS 26.0, and AMOS 24.0. Normality of the continuous data (SRF, PM, HPB, QoL scores) was assessed using the Kolmogorov-Smirnov test and visual inspection of Q-Q plots. The results indicated an approximately normal distribution for all key variables (*p* > 0.05), allowing for parametric testing. Descriptive statistics, including frequencies and percentages, were used to summarize demographic characteristics. Normally distributed continuous variables were presented as means and standard deviations (M ± SD), while non-normally distributed variables were expressed as medians and interquartile ranges. For comparisons across demographic or clinical subgroups (e.g., different age groups, educational levels, or glycemic control categories) within the single study cohort, one-way ANOVA was applied followed by post-hoc comparisons using the Bonferroni correction to adjust for multiple comparisons. Pearson correlation analysis was conducted to examine the relationships among SRF, PM, HPB, and QoL. For all correlations, 95% confidence intervals (CIs) were calculated using Fisher’s z-transformation. Multiple linear regression analysis was performed to identify independent predictors of QoL, with standardized beta coefficients and their 95% CIs reported. A structural equation model (SEM) was constructed using AMOS 24.0 to test the mediating roles of PM and HPB in the relationship between SRF and QoL. Model fit was evaluated using the chi-square/degrees of freedom ratio (CMIN/DF), root mean square error of approximation (RMSEA), goodness-of-fit index (GFI), adjusted goodness-of-fit index (AGFI), Tucker–Lewis index (TLI), comparative fit index (CFI), and incremental fit index (IFI). To assess the significance of mediation effects, the bootstrap method with 5,000 resamples was applied, and statistical significance was set at *p* < 0.05 (two-tailed).

### 2.6 Ethics considerations

The study protocol was approved by the Medical Ethics Committee of Hengyang Central Hospital (Approval No. HYZXYY-2025-06-018, approved in June 2025). Written informed consent was obtained from each participant. All procedures adhered to the Declaration of Helsinki. Participants could refuse or withdraw at any time. Anonymity and confidentiality were guaranteed, and data were used solely for academic purposes.

## 3 Results

### 3.1 Demographic characteristics of T2DM patients

The cohort’s demographic characteristics, including age, sex, BMI, education, and other variables, are summarized in Table 1.

**TABLE 1:** Demographic characteristics and univariate analysis of quality of life among type 2 diabetes mellitus (T2DM) patients (*n* = 432, M ± SD).

Items	Number [*n* (%)]	Quality of life total score	Physical function	Psychological/ spiritual	Social relations	Treatment
Gender						
Male	278 (64.4)	64.11 ± 17.32	27.29 ± 8.61	20.07 ± 7.97	9.70 ± 3.87	7.05 ± 2.87
Female	154 (35.6)	72.34 ± 22.12	31.18 ± 11.91	21.80 ± 8.41	11.26 ± 4.17	8.10 ± 3.41
*t*	–	4.272	3.91	2.082	3.807	3.409
*P*	–	0.000**	0.000**	0.038*	0.000**	0.001**
Age (years)						
18 <45	72 (16.7)	61.03 ± 14.23	25.38 ± 4.50	18.71 ± 7.93	10.10 ± 3.97	6.85 ± 2.82
45 <60	84 (19.4)	66.30 ± 18.60	28.54 ± 9.25	19.99 ± 7.74	10.27 ± 4.08	7.50 ± 3.08
≥60	276 (63.9)	72.54 ± 22.28	31.33 ± 12.28	22.19 ± 8.39	10.99 ± 4.17	8.03 ± 3.38
*F*	–	10.239	9.386	6.273	1.901	4.073
*P*	–	0.000**	0.000**	0.002**	0.151	0.018*
BMI (kg/m2)						
<18.5	72 (16.7)	69.17 ± 20.63	29.25 ± 10.46	21.49 ± 8.30	10.60 ± 4.17	7.83 ± 3.39
18.5~23.9	108 (25.0)	64.17 ± 16.64	26.93 ± 7.97	19.90 ± 8.19	9.94 ± 3.94	7.40 ± 2.81
24.0~27.9	216 (50.0)	69.75 ± 20.99	30.12 ± 11.13	21.19 ± 8.09	10.77 ± 4.03	7.67 ± 3.25
≥28	36 (8.3)	83.53 ± 25.82	37.50 ± 15.03	24.36 ± 9.17	12.78 ± 4.59	8.89 ± 4.07
*F*	–	8.156	8.923	2.691	4.383	1.953
*P*	–	0.000**	0.000**	0.046*	0.005**	0.120
Educational level						
Primary school and below	48 (11.1)	80.17 ± 23.35	35.31 ± 13.71	24.15 ± 7.68	11.83 ± 4.44	8.88 ± 3.52
Junior high school	108 (25.0)	70.23 ± 22.21	30.27 ± 11.93	20.82 ± 8.41	11.06 ± 4.15	8.08 ± 3.52
Senior high school or technical secondary school	168 (38.9)	69.82 ± 20.94	29.45 ± 10.85	21.99 ± 8.28	10.70 ± 4.27	7.68 ± 3.15
College and above	108 (25.0)	63.16 ± 15.78	27.39 ± 7.68	18.97 ± 7.96	9.85 ± 3.58	6.95 ± 2.86
*F*	–	7.903	6.075	5.355	3.036	4.604
*P*	–	0.000**	0.000**	0.001**	0.029*	0.003**
Marital status						
Unmarried	52 (12.0)	64.15 ± 17.25	27.23 ± 8.31	19.23 ± 8.01	10.40 ± 3.82	7.29 ± 3.27
Married	315 (72.9)	68.12 ± 19.38	29.11 ± 10.26	20.98 ± 8.10	10.47 ± 4.03	7.56 ± 3.08
Divorced	27 (6.3)	74.59 ± 25.51	31.89 ± 12.65	22.07 ± 8.40	12.22 ± 4.27	8.41 ± 3.62
Widowed	38 (8.8)	83.53 ± 27.46	37.42 ± 15.27	24.92 ± 9.23	11.95 ± 4.90	9.24 ± 3.99
*F*	–	8.225	8.142	3.775	2.832	3.761
*P*	–	0.000**	0.000**	0.011*	0.038*	0.011*
Living conditions						
Living alone	47 (10.9)	80.47 ± 25.03	35.4 ± 14.21	24.15 ± 8.22	12.15 ± 3.79	8.77 ± 3.89
Living with children	107 (24.8)	67.37 ± 19.00	28.59 ± 9.56	20.48 ± 8.36	10.86 ± 4.20	7.45 ± 3.01
Living with spouse only	205 (47.5)	65.94 ± 17.99	28.05 ± 9.14	20.56 ± 8.13	9.85 ± 3.88	7.48 ± 3.00
Living with parents	49 (11.3)	73.18 ± 25.63	31.51 ± 12.86	21.71 ± 8.42	11.82 ± 4.45	8.14 ± 3.59
Others	24 (5.6)	78.71 ± 22.87	35.54 ± 14.76	22.75 ± 8.33	12.17 ± 4.33	8.25 ± 4.06
*F*	–	6.909	6.968	2.277	5.546	2.059
*P*	–	0.000	0.000	0.060	0.000	0.085
Residence						
Rural	84 (19.4)	79.39 ± 25.42	35.19 ± 14.22	23.48 ± 8.37	11.63 ± 4.52	9.10 ± 3.78
Urban	348 (80.6)	66.99 ± 18.91	28.49 ± 9.65	20.63 ± 8.19	10.48 ± 4.00	7.40 ± 3.04
*F*	–	4.198	4.097	2.848	2.311	3.824
*P*	–	0.000**	0.000**	0.005**	0.021*	0.000**
Employment status						
Employed	79 (18.3)	61.70 ± 13.34	25.52 ± 4.39	19.03 ± 7.69	10.23 ± 3.80	6.92 ± 2.90
Retired	310 (71.8)	69.86 ± 21.43	30.18 ± 11.33	21.27 ± 8.30	10.62 ± 4.15	7.78 ± 3.25
Unemployed	43 (9.9)	80.33 ± 23.14	34.81 ± 14.19	24.49 ± 8.32	12.16 ± 4.33	8.86 ± 3.65
*F*	–	11.897	11.136	6.26	3.312	5.13
*P*	–	0.000**	0.000**	0.002**	0.037*	0.006**
Per capita monthly income (RMB)						
<3,000	156 (36.1)	73.63 ± 22.87	31.87 ± 13.04	22.46 ± 8.20	11.01 ± 4.37	8.29 ± 3.41
3,000 <50,000	204 (47.2)	68.95 ± 20.62	29.50 ± 10.54	20.93 ± 8.39	10.85 ± 4.03	7.67 ± 3.20
≥5000	72 (16.7)	61.54 ± 13.80	26.11 ± 4.75	19.14 ± 7.83	9.61 ± 3.71	6.68 ± 2.83
*F*	–	8.632	7.063	4.202	3.115	6.21
*P*	–	0.000**	0.001**	0.016*	0.045*	0.002**
Medical insurance payment method						
Self-payment	25 (5.8)	66.58 ± 18.68	28.45 ± 9.67	20.41 ± 8.12	10.26 ± 3.92	7.47 ± 3.06
New rural cooperative medical care	83 (19.2)	71.60 ± 21.54	30.31 ± 11.43	22.29 ± 7.96	11.25 ± 4.43	7.75 ± 3.29
Urban medical insurance	275 (63.7)	81.20 ± 27.47	34.84 ± 14.42	25.12 ± 9.65	11.64 ± 4.84	9.60 ± 3.69
Others	49 (11.3)	75.51 ± 24.37	33.88 ± 13.57	21.63 ± 8.53	11.78 ± 4.06	8.22 ± 3.79
*F*	–	6.25	5.614	3.265	3.134	3.791
*P*	–	0.000**	0.001**	0.021*	0.025*	0.011*
Drinking status						
Never	155 (35.9)	65.17 ± 18.32	28.05 ± 9.19	19.93 ± 8.20	9.99 ± 3.81	7.20 ± 2.96
Occasionally	168 (38.9)	70.03 ± 20.90	30.05 ± 10.88	21.44 ± 8.29	10.86 ± 4.16	7.68 ± 3.26
Frequently	109 (25.2)	74.47 ± 23.15	31.87 ± 13.07	22.57 ± 8.24	11.47 ± 4.37	8.56 ± 3.52
*F*	–	6.63	3.987	3.414	4.398	5.712
*P*	–	0.001**	0.019*	0.034*	0.013*	0.004**
Smoking status						
Never	60 (13.9)	60.92 ± 14.56	25.83 ± 5.35	18.82 ± 7.78	9.40 ± 3.89	6.87 ± 2.73
Occasionally	70 (16.2)	69.04 ± 20.35	30.30 ± 11.80	20.84 ± 8.01	10.60 ± 4.15	7.30 ± 3.30
Frequently	302 (69.9)	71.18 ± 21.70	30.46 ± 11.49	21.73 ± 8.39	10.98 ± 4.13	8.00 ± 3.32
*F*	–	6.194	4.592	3.196	3.756	3.803
*P*	–	0.002**	0.011*	0.042*	0.024*	0.023*
Disease duration (years)						
<1	24 (5.6)	58.42 ± 11.65	25.42 ± 6.45	17.42 ± 7.51	9.58 ± 3.57	6.00 ± 2.70
1 <6	132 (30.6)	68.23 ± 20.19	29.53 ± 10.57	20.56 ± 7.98	10.45 ± 4.02	7.70 ± 3.23
6 <10	192 (44.4)	68.35 ± 20.14	29.18 ± 10.37	21.06 ± 8.38	10.46 ± 4.04	7.65 ± 3.11
≥10	84 (19.4)	76.80 ± 23.61	32.85 ± 13.31	23.51 ± 8.30	11.98 ± 4.43	8.46 ± 3.61
*F*	–	6.240	3.712	4.213	3.716	3.792
*P*	–	0.000**	0.012*	0.006**	0.012*	0.010*
Number of comorbidities						
None	28 (6.5)	54.79 ± 15.30	24.43 ± 6.90	15.68 ± 6.56	8.50 ± 4.02	6.18 ± 2.06
1 2	276 (63.9)	69.07 ± 20.69	29.56 ± 10.71	21.08 ± 8.32	10.68 ± 4.12	7.75 ± 3.24
≥3	128 (29.6)	73.33 ± 21.02	31.46 ± 11.96	22.60 ± 8.09	11.23 ± 4.03	8.03 ± 3.43
*F*	–	9.508	4.950	8.338	5.152	3.769
*P*	–	0.000**	0.007**	0.000**	0.006**	0.024*
Treatment modality						
Oral medication	72 (16.7)	70.36 ± 21.20	30.06 ± 11.66	22.31 ± 7.91	10.61 ± 4.22	7.39 ± 3.29
Insulin	168 (38.9)	70.14 ± 21.88	30.08 ± 11.61	21.27 ± 8.37	10.87 ± 4.15	7.92 ± 3.34
Oral + insulin	132 (30.6)	68.65 ± 20.28	29.61 ± 10.41	20.94 ± 8.50	10.27 ± 4.01	7.83 ± 3.20
Others	60 (13.9)	67.87 ± 19.32	29.05 ± 9.87	20.13 ± 8.09	11.28 ± 4.20	7.40 ± 3.16
*F*	–	0.283	0.155	0.803	0.976	0.690
*P*	–	0.837	0.927	0.493	0.404	0.558
Glycemic control status						
Poor	48 (11.1)	84.85 ± 25.99	37.13 ± 14.60	25.44 ± 8.01	12.63 ± 4.37	9.67 ± 3.94
Moderate	108 (25)	68.92 ± 20.86	29.46 ± 10.63	20.76 ± 8.55	10.84 ± 4.28	7.85 ± 3.27
Good	276 (63.9)	66.91 ± 18.75	28.64 ± 9.93	20.61 ± 8.05	10.31 ± 3.94	7.34 ± 3.01
*F*	–	16.186	12.885	7.331	6.678	10.955
*P*	–	0.000**	0.000**	0.001**	0.001**	0.000**
Whether combined with other chronic diseases						
Yes	394 (91.2)	70.36 ± 21.21	30.20 ± 11.31	21.47 ± 8.25	10.82 ± 4.12	7.87 ± 3.27
No	38 (8.8)	59.50 ± 14.07	25.58 ± 5.57	18.21 ± 8.29	9.42 ± 4.03	6.29 ± 2.78
*t*	–	4.311	4.325	2.326	2.009	2.876
*P*	–	0.000**	0.000**	0.020*	0.045*	0.004**
Self-care ability						
Fully independent	132 (30.6)	64.79 ± 17.42	27.30 ± 8.62	20.17 ± 7.79	10.05 ± 3.94	7.27 ± 2.93
Partially independent	252 (58.3)	68.90 ± 20.55	29.63 ± 10.73	20.99 ± 8.45	10.56 ± 4.07	7.72 ± 3.17
Completely dependent	48 (11.1)	84.73 ± 24.51	37.48 ± 14.49	25.00 ± 7.90	13.23 ± 4.05	9.02 ± 4.20
*F*	–	17.442	16.212	6.299	11.294	5.151
*P*	–	0.000**	0.000**	0.002**	0.000**	0.006**

*p < 0.05,

**p < 0.01, *F* values represent test statistics from one-way ANOVA for comparisons across subgroups. *P*-values indicate statistical significance levels for differences among groups. Statistical significance was set at *p* < 0.05.

### 3.2 Scores of QoL, SRF, PM, and HPB among T2DM patients

The total score of QoL was 69.41 ± 20.89, with a mean item score of 2.57 ± 0.77. The total score of SRF was 38.63 ± 12.84, with an overall mean of 2.41 ± 0.80. The total score of PM was 26.12 ± 7.62, and the average score was 3.73 ± 1.09. The total score of HPB was 97.69 ± 20.87, with a mean item score of 3.48 ± 0.95.

All scores are summarized in Table 2.

**TABLE 2:** The scores of quality of life, self-regulatory fatigue, personal mastery, and health-promoting behaviors among type 2 diabetes mellitus (T2DM) patients (*n* = 432, M ± SD).

Items	Number of items	Score range	dimension score	average item score	Ranking
Quality of life total score	27	27 135	69.41 ± 20.89	2.57 ± 0.77	–
Physical function	12	12 60	29.79 ± 11.00	2.48 ± 0.92	4
Psychological/Spiritual	8	8 40	21.18 ± 8.29	2.65 ± 1.04	2
Social relations	4	4 20	10.70 ± 4.13	2.68 ± 1.03	1
Treatment	3	3 15	7.73 ± 3.26	2.58 ± 1.09	3
Self-regulatory fatigue total score	16	18 74	38.63 ± 12.84	2.41 ± 0.80	–
Cognitive control	6	6 25	13.26 ± 6.01	2.65 ± 1.20	2
Behavioral control	5	6 30	12.00 ± 5.78	2.00 ± 0.96	3
Emotional control	5	5 25	13.37 ± 5.26	2.67 ± 1.05	1
Health-promoting behaviors total score	28	28 140	97.69 ± 20.87	3.48 ± 0.95	–
Exercise	7	7 35	24.38 ± 6.61	3.44 ± 1.09	6
Risk avoidance	7	7 35	24.09 ± 7.61	3.51 ± 1.09	3
Stress management	5	5 25	17.56 ± 5.47	3.57 ± 0.98	1
Life appreciation	3	3 15	10.72 ± 2.93	3.46 ± 0.89	5
Health responsibility	3	3 15	10.39 ± 2.68	3.52 ± 1.02	2
Healthy diet	3	3 15	10.55 ± 3.06	3.48 ± 0.95	4
Personal mastery total score	7	8 34	26.12 ± 7.62	3.73 ± 1.09	–

For the Diabetes-Specific Quality of Life Scale (DS-QoLS), a lower score indicates better quality of life.

### 3.3 Univariate analysis of QoL among T2DM patients

Seventeen demographic and clinical variables were associated with QoL in univariate analysis (all *p* < 0.05), including gender, age, BMI, educational level, marital status, living conditions, residence, employment status, per capita monthly income, medical insurance payment method, drinking status, smoking status, disease duration, number of comorbidities, glycemic control status, whether combined with other chronic diseases, and self-care ability (Table 1).

### 3.4 Correlations among QoL, SRF, PM, and HPB among T2DM patients

As shown in Table 3, the total QoL score was positively correlated with SRF (*r* = 0.581, *p* < 0.01, 95% CI [0.52, 0.64]), negatively correlated with PM (*r* = −0.557, *p* < 0.01, 95% CI [−0.62, −0.49]), and negatively correlated with HPB (*r* = −0.613, *p* < 0.01, 95% CI [−0.67, −0.55]). PM and HPB were positively correlated (*r* = 0.607, *p* < 0.01). All subscale correlations were significant in the expected directions.

**TABLE 3:** Correlations among quality of life, self-regulatory fatigue, personal mastery, and health-promoting behaviors among type 2 diabetes mellitus (T2DM) patients (*n* = 432, *r*).

Items	Quality of life total score	Physical function	Psychological/ spiritual	Social relations	Treatment
Self-regulatory fatigue total score	0.581**	0.525**	0.463**	0.455**	0.537**
Cognitive control	0.472**	0.461**	0.422**	0.369**	0.447**
Behavioral control	0.358**	0.336**	0.298**	0.312**	0.328**
Emotional control	0.439**	0.415**	0.386**	0.401**	0.421**
Health-promoting behaviors total score	−0.613**	−0.536**	−0.492**	−0.501**	−0.562**
Exercise	−0.523**	−0.473**	−0.451**	−0.395**	−0.408**
Risk avoidance	−0.508**	−0.482**	−0.463**	−0.376**	−0.411**
Stress management	−0.496**	−0.447**	−0.440**	−0.347**	−0.373**
Life appreciation	−0.479**	−0.439**	−0.438**	−0.361**	−0.382**
Health responsibility	−0.431**	−0.415**	−0.427**	−0.315**	−0.348**
Healthy diet	−0.595**	−0.498**	−0.441**	−0.409**	−0.487**
Personal mastery total score	−0.557**	−0.519**	−0.523**	−0.498**	−0.536**

**P < 0.01.

### 3.5 Multiple linear regression analysis of QoL among T2DM patients

Ten variables were independently associated with QoL in the final regression model: age, educational level, medical insurance payment method, smoking status, glycemic control status, number of comorbidities, self-care ability, SRF, PM, and HPB (*p* < 0.05 for all). The model explained 41.8% of the total variance in QoL (R^2^ = 0.418). Variance inflation factor values ranged from 1.12 to 3.85, indicating no significant multicollinearity. Detailed variable coding and regression coefficients are presented in Tables 4, 5.

**TABLE 4:** Assignment of independent variables (*n* = 432).

Independent variables	Assignment
Age	Dummy variables were set with “18∼ < 45” as the reference: “45∼ < 60” = (0,1,0), “≥60” = (0,0,1)
Educational level	Dummy variables were set with “Primary school and below” as the reference: “Junior high school” = (0,1,0,0), “Senior high school or technical secondary school” = (0,0,1,0), “College and Above” = (0,0,0,1)
Medical insurance payment method	Dummy variables were set with “Self-payment” as the reference: “New rural cooperative medical care” = (0,1,0,0), “Urban medical insurance” = (0,0,1,0), “Others” = (0,0,0,1)
Self-care ability	Dummy variables were set with “Fully independent” as the reference: “Partially independent” = (0,1,0), “Completely dependent” = (0,0,1)
Glycemic control status	Dummy variables were set with “Poor” as the reference: “Moderate” = (0,1,0), “Good” = (0,0,1)
Smoking status	Dummy variables were set with “Never” as the reference: “Occasionally” = (0,1,0), “Frequently” = (0,0,1)
Number of comorbidities	Dummy variables were set with “None” as the reference: “1∼2” = (0,1,0), “≥3” = (0,0,1)
Self-regulatory fatigue total score	Original entry
Personal mastery total score	Original entry
Health-promoting behaviors total score	Original entry

**TABLE 5:** Multiple linear regression analysis of quality of life among type 2 diabetes mellitus (T2DM) patients (*n* = 432).

Dependent variable	Independent variables	Unstandardized coefficient (B)	SE	Standardized coefficient (beta)	95% CI for beta	*t*	*P*	VIF
Quality of life	Constant	3.996	0.265	–	–	15.060	0.000	–
	Age	0.110	0.037	0.108	[0.035, 0.181]	3.001	0.003	2.23
	Educational level	−0.071	0.024	−0.087	[−0.312, −0.193]	−2.940	0.003	1.56
	Medical insurance payment method	−0.097	0.035	−0.088	[−0.351, −0.205]	−2.743	0.006	2.11
	Smoking status	0.126	0.037	0.118	[0.028, 0.176]	3.435	0.001	1.69
	Glycemic control status	−0.137	0.036	−0.121	[−0.417, −0.298]	−3.789	0.000	1.81
	Number of comorbidities	0.084	0.041	0.060	[0.013, 0.208]	2.067	0.039	3.85
	Self-care ability	0.178	0.039	0.142	[0.026, 0.327]	4.554	0.000	1.73
	Self-regulatory fatigue	0.175	0.033	0.181	[0.021, 0.217]	5.262	0.000	2.28
	Personal mastery	−0.203	0.023	−0.285	[−0.035, −0.181]	−8.676	0.000	1.45
	Health-promoting behaviors	−0.394	0.040	−0.363	[−0.442, −0.284]	−9.902	0.000	1.12

*R* = 0.656, *R*^2^ = 0.431, adjusted *R*^2^ = 0.418, *F* = 112.518, *p* < 0.001.

### 3.6 Mediating roles of PM and HPB between SRF and QoL

**FIGURE 2:**
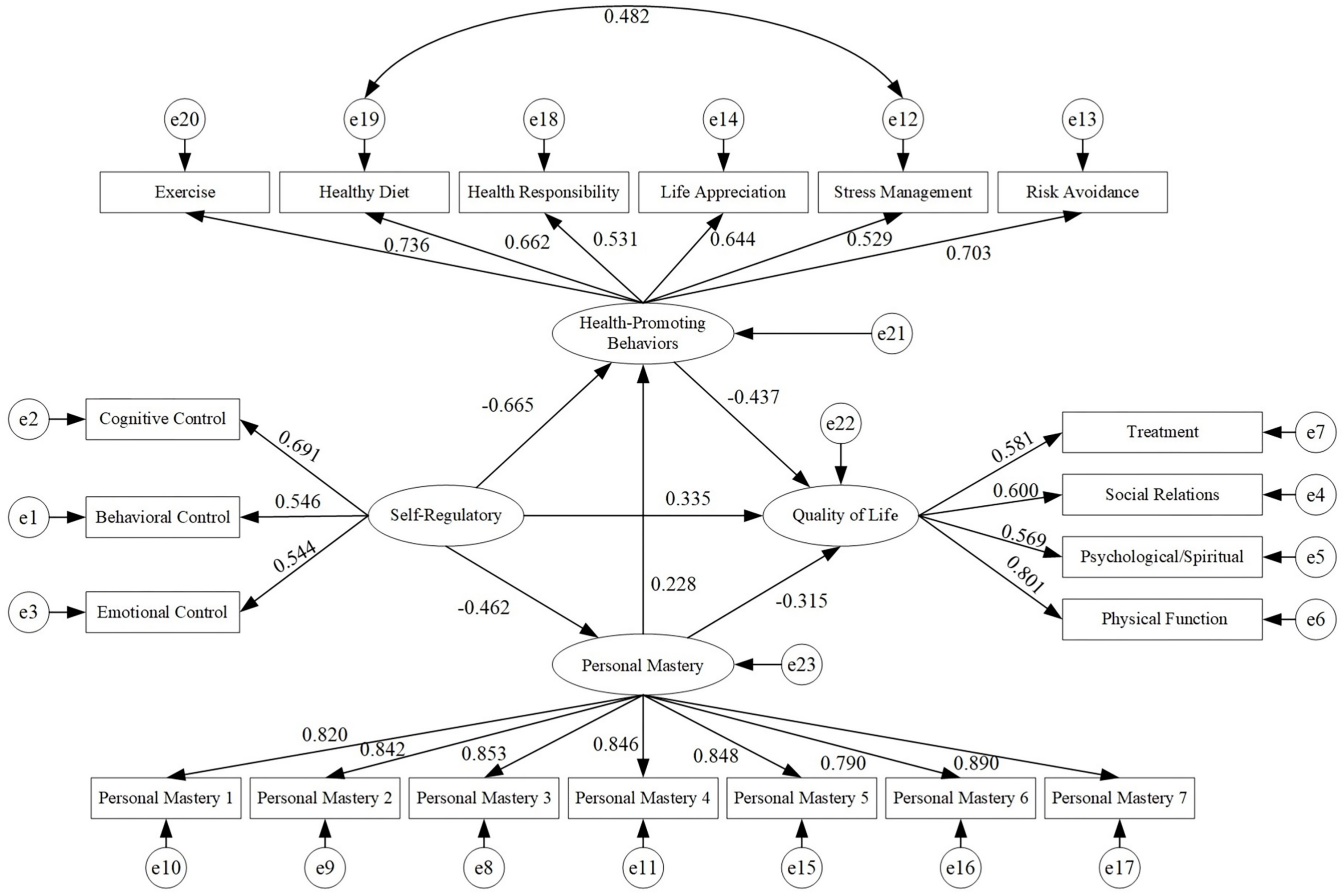
Multiple mediating effect model of personal mastery and health-promoting behaviors in the relationship between self-regulatory fatigue and quality of life.

The structural equation model showed good fit: χ^2^/df = 1.906, RMSEA = 0.046, GFI = 0.932, AGFI = 0.912, TLI = 0.963, CFI = 0.968, IFI = 0.969 (Figure 2).

Bootstrap analysis indicated that the total effect of SRF on QoL was 0.845, consisting of a direct effect of 0.362 and a total indirect effect of 0.483. The total indirect effect accounted for 57.2% of the total effect.

The specific indirect effects via PM only, HPB only, and the PM→HPB chain were 0.146 (17.3% of total effect), 0.291 (34.4%), and 0.046 (5.4%), respectively. Relative to the total indirect effect (0.483), these pathways accounted for 28.7%, 44.6%, and 11.3%, respectively (Table 6).

**TABLE 6:** Decomposition of direct, indirect, and total effects in the mediation model (*n* = 432).

Effect type	Pathway	Effect size	Proportion of total effect (%)	Proportion of total indirect effect (%)
Direct effect	SRF → QoL	0.362	42.8%	–
Total Indirect Effect	SRF → PM/HPB → QoL	0.483	57.2%	100.0%
Specific indirect effects	SRF → PM → QoL	0.146	17.3%	30.2%
	SRF → HPB → QoL	0.291	34.4%	60.2%
	SRF → PM → HPB → QoL (Chain)	0.046	5.4%	9.5%
Total effect	SRF → QoL	0.845	100.0%	–

All indirect effects were significant at *p* < 0.01 (two-tailed), as determined by bias-corrected bootstrap 95% confidence intervals. Percentages are rounded and may not sum to 100% due to rounding. The proportions of specific indirect effects relative to the total indirect effect (e.g., 28.7%, 44.6%, 11.3% as reported in the text) are calculated as (specific indirect effect/total indirect effect). Slight discrepancies between the values in this table and those in the text are due to rounding for presentation clarity; the text reports the exact calculated percentages.

## 4 Discussion

This study examined QoL and its associated factors in T2DM patients, with a primary focus on the mediating roles of PM and HPB between SRF and QoL. The results revealed a moderate level of QoL in this sample, with ten independent predictors identified. Most importantly, the structural equation model demonstrated that SRF was associated with lower QoL primarily through indirect pathways, with the serial mediation of PM→HPB accounting for 11.3% of the total effect. These findings position PM as a critical “meta-resource” that, when depleted by SRF, compromises HPB and ultimately reduces QoL.

### 4.1 Status and core predictors of QoL

The overall QoL among T2DM patients was moderate, consistent with previous reports (22–24). Participants reported relatively better QoL in the social relations and psychological/spiritual domains, but poorer QoL in the treatment and physical function domains. The lower physical function score directly reflects the core pathophysiology of T2DM, where chronic hyperglycemia and insulin resistance lead to end-organ damage, neuropathy, and fatigue, significantly limiting physical capacity.

Ten independent predictors explained 41.8% of the variance in QoL, including age, educational level, medical insurance, smoking status, glycemic control, number of comorbidities, self-care ability, SRF, PM, and HPB. Among these, the latter three psychological and behavioral variables demonstrated the strongest associations. While the associations for demographic and clinical variables (e.g., age, education, comorbidities) align with established literature (25–31), the inclusion of SRF, PM, and HPB in a single model provides new evidence for their simultaneous contribution to QoL beyond traditional risk factors.

### 4.2 Correlations among QoL, SRF, PM, and HPB

#### 4.2.1 SRF is positively correlated with QoL

Consistent with prior work (32, 33), the present study identified a significant positive correlation between SRF and QoL scores (*r* = 0.581, *p* < 0.01), indicating that greater self-regulatory fatigue is associated with poorer quality of life. This association is underpinned by multiple interrelated mechanisms. Pathophysiologically, the tension between subjective volition and physiological limitations may exacerbate diabetes-related metabolic dysregulation and fatigue. Psychologically, persistent regulatory demands coupled with ineffective coping strategies can precipitate negative emotions and reduced self-efficacy, directly impairing wellbeing. Behaviorally, fatigue-driven cognitive biases may lead to maladaptive self-management (e.g., excessive rest or inappropriate dietary adjustments), creating a vicious cycle that further compromises glycemic control and overall QoL (33).

#### 4.2.2 PM is negatively correlated with QoL

As hypothesized, PM showed a significant negative correlation with QoL (*r* = −0.557, *p* < 0.01), reflecting that higher personal mastery is associated with better QoL. This finding aligns with Deng (34) and can be explained from multiple dimensions. Physically, greater mastery promotes autonomous, standardized disease management (e.g., glucose monitoring and treatment adherence), which improves metabolic control and reduces physical discomfort. Psychologically, a strong sense of control fosters self-efficacy and alleviates anxiety and depression related to poor disease outcomes. Socially, patients with higher mastery are more likely to communicate proactively with family and healthcare providers and engage in social activities, thereby strengthening support systems and reducing disease-related isolation. These integrated benefits across physical, psychological, and social domains ultimately contribute to a higher perceived QoL.

#### 4.2.3 HPB is negatively correlated with QoL

Similarly, HPB was negatively correlated with QoL (*r* = −0.613, *p* < 0.01), indicating that more frequent engagement in health-promoting behaviors predicts a substantially better QoL, consistent with Zhang (35). Active performance of healthy behaviors such as medication adherence, dietary regulation, and regular exercise directly stabilizes blood glucose and reduces complication risks, leading to improved physiological functioning. Concurrently, proactive health management fosters a sense of control and reduces feelings of helplessness, thereby enhancing psychological wellbeing. Additionally, consistent HPB reduces healthcare utilization and associated economic burdens, indirectly improving life satisfaction. These direct and indirect benefits across physical, psychological, and socioeconomic domains underscore the critical role of HPB in preserving QoL among T2DM patients.

### 4.3 The central role of mediation pathways

The primary novel contribution of this study is the empirical test of a hierarchical depletion model linking SRF to QoL through PM and HPB. The structural equation model exhibited good fit (χ^2^/df = 1.906, RMSEA = 0.046), and bootstrap analysis revealed that the total indirect effect (0.483) accounted for 57.2% of the total effect of SRF on QoL, substantially exceeding the direct effect (0.362).

(1) SRF → PM → QoL (28.7% of total effect): This pathway supports the Conservation of Resources (COR) theory (36), which posits that individuals strive to protect limited self-regulatory resources. In T2DM, sustained self-management demands excessively consume these resources, leading to SRF. This depletion directly undermines PM—an individual’s global belief in their ability to control diabetes-related life events. When PM is diminished, patients lose confidence in their capacity to manage their condition, which impairs treatment adherence and social participation, ultimately reducing QoL. This finding extends previous work by positioning PM as the psychological mechanism through which fatigue erodes well-being.

(2) SRF → HPB → QoL (44.6% of total effect): This pathway reflects a more immediate behavioral consequence of resource depletion. According to the Theory of Planned Behavior (37), SRF weakens perceived behavioral control. When patients feel exhausted, they perceive themselves as unable to sustain healthy behaviors such as regular exercise or dietary monitoring. This reduced self-efficacy leads directly to disengagement from HPB, which in turn worsens glycemic control, increases psychological distress, and lowers QoL. The larger magnitude of this pathway (44.6%) compared to the PM-only pathway (28.7%) suggests that the direct behavioral impact of fatigue may be more salient than the generalized psychological belief system.

(3) SRF → PM → HPB → QoL (11.3% of total effect): The significant serial mediation provides the strongest support for our proposed hierarchical model. This chain pathway posits that SRF first erodes the higher-order meta-resource of PM, and this erosion subsequently leads to a decline in specific behavioral resources (HPB) (38). The positive correlation between PM and HPB (*r* = 0.607) is not a collinearity issue but a conceptual prerequisite for this cascade. Without this covariation, a sequential effect would be impossible. Clinically, this means that interventions that only target HPB (e.g., exercise programs) without addressing patients’ underlying sense of mastery may yield only temporary improvements (39). Once the intervention ends, low PM may cause patients to revert to previous behavioral patterns.

### 4.4 Clinical implications

These findings suggest a hierarchical intervention strategy. Primary interventions should target SRF through energy conservation techniques, structured diabetes education that reduces decision fatigue, and mindfulness-based stress reduction. Secondary interventions should directly build PM using empowerment-based education, collaborative goal setting, and individualized feedback to enhance patients’ confidence in controlling diabetes-related life events. For patients with intractably low PM, behavioral activation (HPB) may serve as a “bottom-up” strategy, where small behavioral successes gradually restore a sense of mastery. The 11.3% chain mediation effect, while modest, indicates that addressing PM first may amplify the sustainability of HPB interventions.

### 4.5 Limitations

Several limitations must be acknowledged. (1) Sampling bias: Convenience sampling from tertiary hospitals may overestimate PM and QoL, limiting generalizability to primary care settings. (2) Causal inference: The cross-sectional design precludes definitive causal conclusions; longitudinal studies are required. (3) Unexplained variance: The model explained 41.8% of QoL variance; other factors such as depression, diabetes distress, and social support were not measured. (4) Instrument validation: Although all scales showed good internal consistency, their full psychometric properties in this specific population require further confirmation. (5) Oversampling: Our sample (*n* = 432) exceeded the required size (*n* = 107), which enhances power but may also detect trivial effects; findings require replication.

## 5 Conclusion

Self-regulatory fatigue is associated with lower QoL in T2DM patients, largely through indirect pathways involving PM and HPB. The serial mediation (SRF → PM → HPB → QoL) provides novel empirical support for a hierarchical resource depletion model. Clinically, strengthening personal mastery through empowerment-based education, goal setting, and individualized feedback may enhance patients’ confidence in diabetes self-management, promote sustained engagement in healthy behaviors, and ultimately improve QoL. Interventions targeting self-regulatory resources and personal mastery should be prioritized.

## Data Availability

The original contributions presented in this study are included in this article/supplementary material, further inquiries can be directed to the corresponding authors.
